# Systemic immune-inflammation index associated with functional outcomes after endovascular thrombectomy in anterior circulation acute ischemic stroke

**DOI:** 10.3389/fneur.2025.1663299

**Published:** 2025-10-22

**Authors:** Li Han, Tao Miao, Le-Yi Zheng, Xiao-Fei Hu, Jia-Wei Zhong

**Affiliations:** ^1^Department of Neurology, Taizhou Hospital of Zhejiang Province, Affiliated to Wenzhou Medical University, Linhai, Zhejiang, China; ^2^Department of Nursing, Taizhou Hospital of Zhejiang Province, Affiliated to Wenzhou Medical University, Linhai, Zhejiang, China; ^3^College of Nursing, Wenzhou Medical University, Wenzhou, Zhejiang, China

**Keywords:** systemic immune-inflammation index, endovascular thrombectomy, anterior circulation stroke, functional outcome, inflammation

## Abstract

**Background:**

Many patients undergoing endovascular thrombectomy (EVT) for anterior circulation acute ischemic stroke experience poor outcomes despite successful recanalization. The systemic immune-inflammation index (SII) integrates multiple inflammatory pathways. We aimed to evaluate the association between SII and clinical outcomes in anterior circulation stroke patients undergoing EVT.

**Methods:**

This retrospective study included 741 consecutive patients who underwent EVT for anterior circulation stroke at a tertiary center between January 2021 and December 2024. SII was calculated as platelet count × neutrophil count/lymphocyte count within 24 h. The primary outcome was poor functional outcome (modified Rankin Scale 3–6) at 3 months. The safety endpoint was symptomatic intracranial hemorrhage (sICH). Associations were examined using multivariable logistic regression and cubic spline analyses.

**Results:**

The median SII was 1,247.9 [IQR: 804.9–2127.1]. Poor functional outcome occurred in 317 (42.8%) patients. After adjustment, log-transformed SII was independently associated with poor functional outcome (OR 1.428, 95% CI 1.159–1.759, *P* < 0.001). Patients in the highest SII tertile (>1,809) had significantly higher odds of poor outcome vs. lowest tertile ( ≤ 928.25) (OR 1.73, 95% CI 1.18–2.52, *P* = 0.005), with significant trend across tertiles (*P* for trend = 0.005). However, SII showed no association with symptomatic intracranial hemorrhage (OR 1.32, 95% CI 0.84–2.07, *P* = 0.235). The SII-outcome association was consistent across subgroups. Restricted cubic spline analysis confirmed a linear dose-response relationship (*P* for non-linearity = 0.173).

**Conclusions:**

In anterior circulation stroke patients undergoing EVT, elevated SII is independently associated with poor functional outcome but not symptomatic hemorrhagic complications.

## Introduction

Acute ischemic stroke (AIS) remains a leading cause of mortality and long-term disability worldwide, with large vessel occlusion (LVO) strokes accounting for the most severe cases and poorest outcomes (1). Endovascular thrombectomy (EVT) has revolutionized the treatment of LVO-related AIS, demonstrating significant improvements in functional outcomes compared to medical management alone (2, 3). The evidence for EVT efficacy is particularly robust for anterior circulation LVO, with multiple randomized controlled trials establishing clear benefits (2, 3). Recent advances have further expanded EVT indications to include patients with large ischemic cores and posterior circulation strokes (4–8). However, despite high rates of successful recanalization approaching 90%, many patients still experience poor functional outcomes, with more than half failing to achieve functional independence (modified Rankin Scale [mRS] 0–2) at 3 months (9–11). This discrepancy between angiographic success and clinical outcomes underscores the need for reliable biomarkers to identify patients at risk for poor outcomes and guide personalized treatment strategies.

The inflammatory response plays a pivotal role in the pathophysiology of ischemic stroke and significantly influences clinical outcomes (12). The systemic immune-inflammation index (SII), calculated as platelet count × neutrophil count/lymphocyte count, represents a composite inflammatory marker that integrates multiple components of the inflammatory cascade. Unlike two-component ratios such as NLR and PLR, SII integrates three key components: neutrophils (representing innate immunity and tissue damage), lymphocytes (adaptive immunity and immunosuppression), and platelets (thrombosis and inflammation), theoretically capturing the complex interplay between inflammation and thrombosis more comprehensively than simpler indices (13).

SII has been widely investigated as a prognostic marker in oncology and cardiovascular diseases (13–15). In cerebrovascular disease, elevated SII levels have been associated with poor outcomes in various stroke subtypes (16–18). Studies in EVT-treated patients have demonstrated associations between SII and multiple adverse outcomes, including malignant cerebral edema, hemorrhagic transformation, and poor functional prognosis (19–23). However, most existing studies have been limited by relatively small sample sizes, single outcome measures, or lack of comprehensive safety endpoint assessment.

Despite growing interest in SII as a prognostic biomarker, several important knowledge gaps remain. First, the relationship between SII and both efficacy and safety outcomes within the same cohort has not been comprehensively evaluated. Second, whether SII maintains its prognostic value across different patient subgroups and treatment modalities remains unclear. Third, the shape of the dose-response relationship between SII and clinical outcomes has not been well characterized. Finally, potential effect modifiers of the SII-outcome association, such as stroke etiology or bridging thrombolysis, require further investigation.

Therefore, this study aimed to comprehensively evaluate the association between admission SII levels and clinical outcomes in patients with anterior circulation AIS undergoing EVT. We specifically sought to: (1) determine the association between SII and 3-month functional outcomes; (2) assess whether SII is associated with hemorrhagic complications; (3) characterize the dose-response relationship between SII and outcomes; and (4) identify potential effect modifiers through subgroup analyses. With a relatively large sample of 741 patients from a single center, our study provides a unique opportunity to address these knowledge gaps while minimizing inter-center variability in treatment protocols and outcome assessment.

## Methods

### Study design and data source

This retrospective observational cohort study was conducted at Taizhou Hospital of Zhejiang Province, a tertiary care center serving the Taizhou region of Zhejiang Province, China. We retrospectively analyzed data from consecutive patients with acute ischemic stroke who underwent endovascular thrombectomy between January 2021 and December 2024. The study protocol was approved by the institutional review board of Taizhou Hospital of Zhejiang Province (Registration number: K20181204), and the requirement for informed consent was waived due to the retrospective nature of the study.

The selection of SII as our primary inflammatory biomarker was based on its comprehensive integration of multiple inflammatory pathways and demonstrated prognostic value across various pathological conditions. Rather than conducting comparative analyses with other inflammatory indices, which would substantially expand the study scope, we focused on thoroughly investigating SII's prognostic value through comprehensive dose-response relationships, subgroup analyses, and safety endpoint assessments.

All clinical data were systematically collected and maintained in a standardized stroke database, including baseline demographics, medical history, laboratory parameters, imaging findings, treatment variables, and functional outcomes. Data quality was ensured through regular monitoring and validation processes.

### Patient selection

Inclusion Criteria: Patients were included if they met the following criteria: (1) age ≥ 18 years; (2) clinical diagnosis of acute anterior circulation ischemic stroke confirmed by neuroimaging; (3) underwent endovascular thrombectomy; and (4) availability of complete laboratory data required for SII calculation within 24 h after the procedure. Exclusion Criteria: Exclusion criteria were: (1) unsuccessful vascular recanalization or pre-procedure recanalization (defined as failure to achieve mTICI grade 2b or 3 flow) (9); (2) missing 3-month functional outcome assessment; (3) patients with multiple endovascular thrombectomy procedures within 1 year; (4) incomplete laboratory data essential for SII calculation, including neutrophil count, lymphocyte count, or platelet count; and (5) posterior circulation stroke. The exclusion of posterior circulation strokes was based on well-established anatomical and pathophysiological differences between vascular territories. The posterior circulation demonstrates distinct ischemic susceptibility patterns, with brain stem and cerebellar regions showing greater resistance to ischemic injury compared to anterior circulation regions (24, 25). Clinical presentations of posterior circulation strokes are often atypical and variable, leading to diagnostic challenges and increased heterogeneity in patient selection and outcome assessment (25, 26). Additionally, hemorrhagic complications after revascularization therapies differ significantly between circulations, with notably lower rates in posterior circulation strokes (25, 27). Given these fundamental differences, we focused on anterior circulation strokes to enhance internal validity and reduce treatment heterogeneity. Anterior circulation acute ischemic stroke was defined as involving the internal carotid artery (ICA) system, including the middle cerebral artery (MCA) and its branches, the anterior cerebral artery (ACA) and its branches, ICA territory infarctions, and clinically significant anterior choroidal artery infarctions.

## Study endpoints

### Primary endpoints

The primary endpoint was poor functional outcome at 3 months, defined as modified Rankin Scale (mRS) (11) score of 3–6. The mRS assessment was performed by trained neurologists or stroke nurses through structured telephone interviews or clinic visits at 3 months post-procedure. Outcome assessors were blinded to the SII values and other study variables when feasible.

### Safety endpoints

The safety endpoint was symptomatic intracranial hemorrhage (sICH), defined according to international standards as any hemorrhagic transformation combined with either a ≥4-point increase in NIHSS score compared to the immediate pre-deterioration neurological status or requirement for neurosurgical intervention (28). sICH was selected as the primary safety endpoint due to its greater clinical relevance and standardized definition across major EVT trials.

### Calculation of systemic immune-inflammation index

The SII was calculated using the formula: SII = platelet count ( × 10^9^/L) × neutrophil count ( × 10^9^/L)/lymphocyte count ( × 10^9^/L). Blood samples were obtained within 24 h after endovascular thrombectomy, following standardized laboratory protocols. All laboratory measurements were performed using automated analyzers with established quality control procedures.

### Data collection and variable definitions

Baseline demographic and clinical characteristics were systematically recorded, including age, sex, medical comorbidities (hypertension, diabetes mellitus, atrial fibrillation, and previous stroke), smoking status, and laboratory parameters (complete blood count with differential, hemoglobin, serum creatinine, blood glucose, lipid profile, and D-dimer). Blood samples for SII calculation were obtained within 24 h after endovascular thrombectomy. Stroke severity was assessed using NIHSS at admission. Imaging characteristics included ASPECTS and occlusion site. Treatment variables encompassed puncture-to-reperfusion time, use of intravenous thrombolysis, and stroke etiology (TOAST classification).

Stroke severity was assessed using the National Institutes of Health Stroke Scale (NIHSS) at admission. Imaging characteristics included Alberta Stroke Program Early CT Score (ASPECTS) and occlusion site. Treatment variables encompassed puncture-to-reperfusion time (PRT), use of intravenous thrombolysis, reperfusion outcomes (modified Thrombolysis in Cerebral Infarction [mTICI] (9) scale), and stroke etiology according to Trial of Org 10172 in Acute Stroke Treatment (TOAST) classification.

### Mechanical thrombectomy procedure

All procedures were performed by experienced neurointerventionalists according to institutional protocols. Successful recanalization was defined as achieving modified Thrombolysis in Cerebral Infarction (mTICI) (9) grade 2b or 3 flow.

### Statistical analysis

Baseline characteristics were presented according to SII tertiles. Continuous variables were expressed as mean ± standard deviation for normally distributed data or median (interquartile range [IQR]) for non-normally distributed data. Categorical variables were presented as counts (percentages). Differences across tertiles were compared using one-way ANOVA or Kruskal-Wallis test for continuous variables and chi-squared test or Fisher's exact test for categorical variables, as appropriate.

Due to the skewed distribution of SII values, logarithmic transformation (log_SII) was applied when SII was analyzed as a continuous variable. Associations between SII and outcomes were examined using logistic regression models with three levels of adjustment: Model 1 (unadjusted), Model 2 (adjusted for age and sex), and Model 3 (fully adjusted).

The fully adjusted model (Model 3) included age, gender, smoking, hypertension, diabetes, atrial fibrillation, thrombolysis, ASPECTS, and puncture-to-reperfusion time (PRT). Variable selection was guided by directed acyclic graph (DAG) principles (29, 30) to distinguish between confounders and potential mediators. Baseline NIHSS was conceptualized as a mediator on the causal pathway (patient characteristics → stroke severity → inflammatory response → functional outcomes) rather than a confounder. Adjusting for mediators can introduce over adjustment bias by blocking causal pathways and underestimating total effects (31). Therefore, baseline NIHSS was excluded from the primary model to estimate the total effect of SII. In contrast, ASPECTS was retained as it represents baseline anatomical burden (early ischemic changes) that is a common cause of both SII levels and functional outcomes, fulfilling the definition of a confounder. To ensure transparency and assess robustness, sensitivity analyses including baseline NIHSS were performed (Model 4, Supplementary Table S1).

In addition to continuous analysis, SII was also analyzed as categorical tertiles based on the original values, with the lowest tertile (T1) serving as the reference group. *P* for trend across tertiles was calculated by modeling tertiles as a continuous variable.

Subgroup analyses examined the association between log-transformed SII and poor functional outcome across patient characteristics using fully adjusted logistic regression models (Model 3 variables) with interaction terms to assess effect modification. For each subgroup, we: (1) ran separate fully adjusted models to obtain subgroup-specific odds ratios and *P*-values; (2) tested interaction terms to assess whether the SII-outcome association differed significantly across subgroup categories. Both individual subgroup *P*-values and interaction *P*-values are reported, with the latter being the primary test for effect modification.

Restricted cubic spline analyses were performed to assess the shape of the association between log (SII) and outcomes, using three knots at the 10th, 50th, and 90th percentiles. The reference value was set at the 10th percentile (log_SII = 6.224).

No formal a priori sample size calculation was performed for this retrospective study. All consecutive eligible patients during the study period were included to maximize statistical power and minimize selection bias. The final sample of 741 patients demonstrated adequate statistical power, as evidenced by the significant findings with narrow confidence intervals.

All analyses were performed using R version 4.1.0 (R Foundation for Statistical Computing, Vienna, Austria). A two-sided *P* value < 0.05 was considered statistically significant.

## Results

### Study population

**Figure 1:**
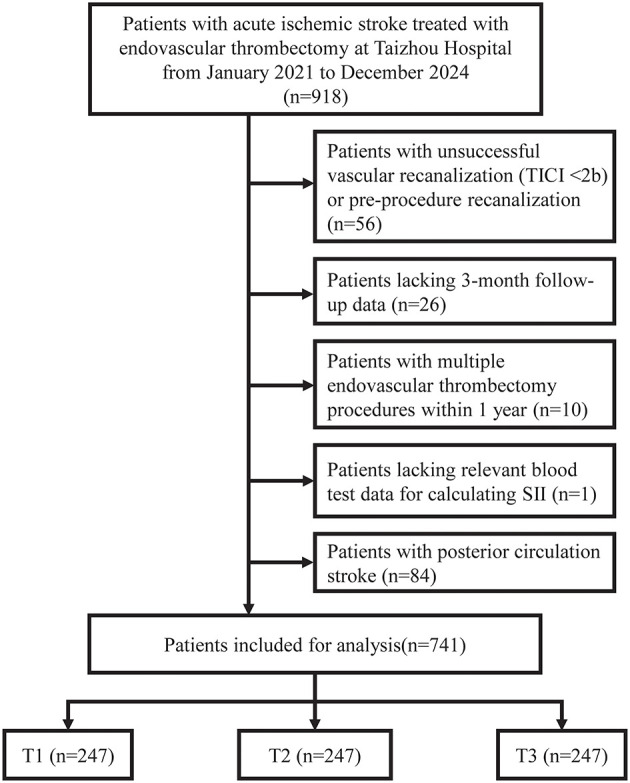
Flowchart of patient selection. Patient selection flowchart. TICI, Thrombolysis in Cerebral Infarction; SII, systemic immune-inflammation index.

Among 918 patients with acute ischemic stroke who underwent endovascular thrombectomy between January 2021 and December 2024, 741 patients met the inclusion criteria and were included in the final analysis (Figure 1). A total of 177 patients were excluded: 56 due to unsuccessful vascular recanalization (TICI < 2b) or pre-procedure recanalization, 84 with posterior circulation stroke, 26 lacking 3-month follow-up data, 10 with multiple endovascular thrombectomy procedures within 1 year, and 1 lacking relevant blood test data for SII calculation.

### Baseline characteristics

The median SII was 1,247.9 [IQR: 804.9–2,127.1]. Patients were stratified into tertiles: T1 ( ≤ 928.25), T2 (928.26–1,809), and T3 (>1,809), each *n* = 247.

Higher SII tertiles showed progressively higher neutrophil counts (5.15 vs. 7.04 vs. 9.69 × 10^9^/L) and platelet counts (167.01 vs. 195.45 vs. 229.98 × 10^9^/L), but lower lymphocyte counts (1.49 vs. 1.10 vs. 0.79 × 10^9^/L, all *P* < 0.001). Clinical presentation severity increased with SII tertiles, as evidenced by higher admission NIHSS scores (mean: 12.98 vs. 13.72 vs. 15.34, *P* < 0.001) and a greater proportion of patients with NIHSS ≥15 (36.8% vs. 38.9% vs. 53.4%, *P* < 0.001). Other baseline characteristics including age, gender distribution, vascular risk factors, and procedural variables were generally balanced across tertiles, except for serum creatinine (*P* < 0.001), total cholesterol (*P* = 0.02), triglycerides (*P* = 0.02), and D-dimer levels (*P* < 0.001), which showed significant differences. Details are in Table 1.

**Table 1:** Baseline characteristics and clinical parameters of patients with anterior circulation acute ischemic stroke undergoing endovascular thrombectomy according to SII tertiles (*n* = 741).

**Variables**	**Overall (*n =* 741)**	**T1 (*n* = 247)**	**T2 (*n* = 247)**	**T3 (*n* = 247)**	***P*-value**
Systemic immune-inflammation index (SII) (median [IQR])	1,247.9 [804.9, 2,127.1]	661.1 [466.7, 801.2]	1,247.9 [1,094.2, 1,462.2]	2,674.7 [2,142.2, 3,343.5]	< 0.001
Log-transformed SII [mean (SD)]	7.16 (0.76)	6.36 (0.45)	7.15 (0.18)	7.98 (0.44)	< 0.001
Neutrophil absolute count (median [IQR]), × 10^9^/L	7.00 [5.30, 9.20]	5.15 [4.20, 6.12]	7.04 [5.76, 8.20]	9.69 [7.80, 11.78]	< 0.001
Lymphocyte absolute count [mean (SD)], × 10^9^/L	1.12 (0.56)	1.49 (0.63)	1.10 (0.45)	0.79 (0.33)	< 0.001
Platelet count [mean (SD)], × 10^9^/L	197.48 (70.60)	167.01 (48.92)	195.45 (57.64)	229.98 (85.34)	< 0.001
**Demographic variables**					
Age [mean (SD)]	69.38 (11.85)	70.20 (11.46)	69.55 (11.55)	68.40 (12.49)	0.231
Gender, Female (%)	298 (40.2)	106 (42.9)	99 (40.1)	93 (37.7)	0.49
**Clinical presentation**					
Admission NIHSS score [mean (SD)]	14.01 (6.59)	12.98 (6.29)	13.72 (6.28)	15.34 (6.97)	< 0.001
Admission NIHSS score ≥15 (%)	319 (43.0)	91 (36.8)	96 (38.9)	132 (53.4)	< 0.001
ASPECT [mean (SD)]	8.51 (1.80)	8.58 (1.76)	8.43 (1.83)	8.51 (1.81)	0.65
ASPECT>7 (%)	573 (77.3)	198 (80.2)	186 (75.3)	189 (76.5)	0.406
**Medical history/comorbidities**					
Smoking (%)	175 (23.6)	60 (24.3)	55 (22.3)	60 (24.3)	0.829
Hypertension (%)	438 (59.1)	136 (55.1)	161 (65.2)	141 (57.1)	0.053
Arterial fibrillation (%)	310 (41.8)	109 (44.1)	101 (40.9)	100 (40.5)	0.667
Diabetes (%)	139 (18.8)	51 (20.6)	47 (19.0)	41 (16.6)	0.51
Previous stroke history (%)	161 (21.7)	52 (21.1)	46 (18.6)	63 (25.5)	0.17
**Endovascular treatment**					
Intravenous thrombolysis (%)	178 (24.0)	51 (20.6)	67 (27.1)	60 (24.3)	0.24
TOAST (%)					0.549
Large Artery	401 (54.1)	128 (51.8)	137 (55.5)	136 (55.1)	
Cardioembolic	319 (43.0)	109 (44.1)	106 (42.9)	104 (42.1)	
Other/Unknown	21 (2.8)	10 (4.0)	4 (1.6)	7 (2.8)	
Puncture to reperfusion time (median [IQR]), min	63 [43, 99]	60 [41, 90]	65 [44.5, 101.5]	65[43.5, 100]	0.262
**Laboratory results**					
Hemoglobin (median [IQR]), g/dL	124.34 (19.45)	124.52 (21.06)	124.81 (18.45)	123.70 (18.82)	0.805
White blood cell count (median [IQR]), 10^9^/L	8.64 [7.00, 10.57]	7.28 [5.96, 8.80]	8.66 [7.10, 10.00]	10.37 [8.60, 12.70]	0.1
Serum creatinine (median [IQR]),μmol/L	66.00 [54.00, 79.00]	66.00 [54.00, 79.00]	64.00 [54.00, 80.00]	67.00 [55.00, 78.00]	< 0.001
Blood glucose [mean (SD)], mmol/L	7.37 (2.44)	7.27 (2.38)	7.38 (2.38)	7.45 (2.55)	0.708
Total cholesterol (median [IQR]), mmol/L	0.96 [0.72, 1.46]	1.10 [0.74, 1.48]	0.94 [0.74, 1.60]	0.87 [0.67, 1.27]	0.002
LDL cholesterol [mean (SD)], mmol/L	2.42 (4.47)	2.29 (0.77)	2.32 (0.75)	2.66 (7.72)	0.601
Triglycerides (median [IQR]), mmol/L	0.96 [0.72, 1.46]	1.10 [0.74, 1.48]	0.94 [0.74, 1.60]	0.87 [0.67, 1.27]	0.002
D-dimer (median [IQR]), mg/L	1.17 [0.60, 2.23]	0.82 [0.50, 1.83]	1.29 [0.64, 2.22]	1.37 [0.67, 2.66]	< 0.001
**Clinical outcomes**					
Poor functional outcome (mRS 3–6) at 3 months (%)	317 (42.8)	92 (37.2)	105 (42.5)	120 (48.6)	0.039
Length of hospital stay [mean (SD)], days	11.9 (9.0)	11.8 (10.1)	11.7 (6.8)	12.4 (9.9)	0.671
ICU length of stay [mean (SD)], days	3.3 (7.5)	3.2 (7.6)	3.1 (6.2)	3.5 (8.4)	0.871
Symptomatic intracranial hemorrhage (%)	39 (5.3%)	13 (5.3%)	8 (3.2%)	18 (7.3%)	0.148

SII tertile ranges: T1: ≤ 928.25 (*n* = 247), T2: 928.26–1,809 (*n* = 247), T3: >1,809 (*n* = 247). SII, systemic immune-inflammation index; IQR, interquartile range; NIHSS, National Institutes of Health Stroke Scale; ASPECT, Alberta Stroke Program Early CT Score; TOAST, Trial of Org 10172 in Acute Stroke Treatment; mRS, modified Rankin Scale; ICU, intensive care unit; PRT, puncture to reperfusion time.

### Primary outcome: poor functional outcome at 3 months

**Figure 2:**
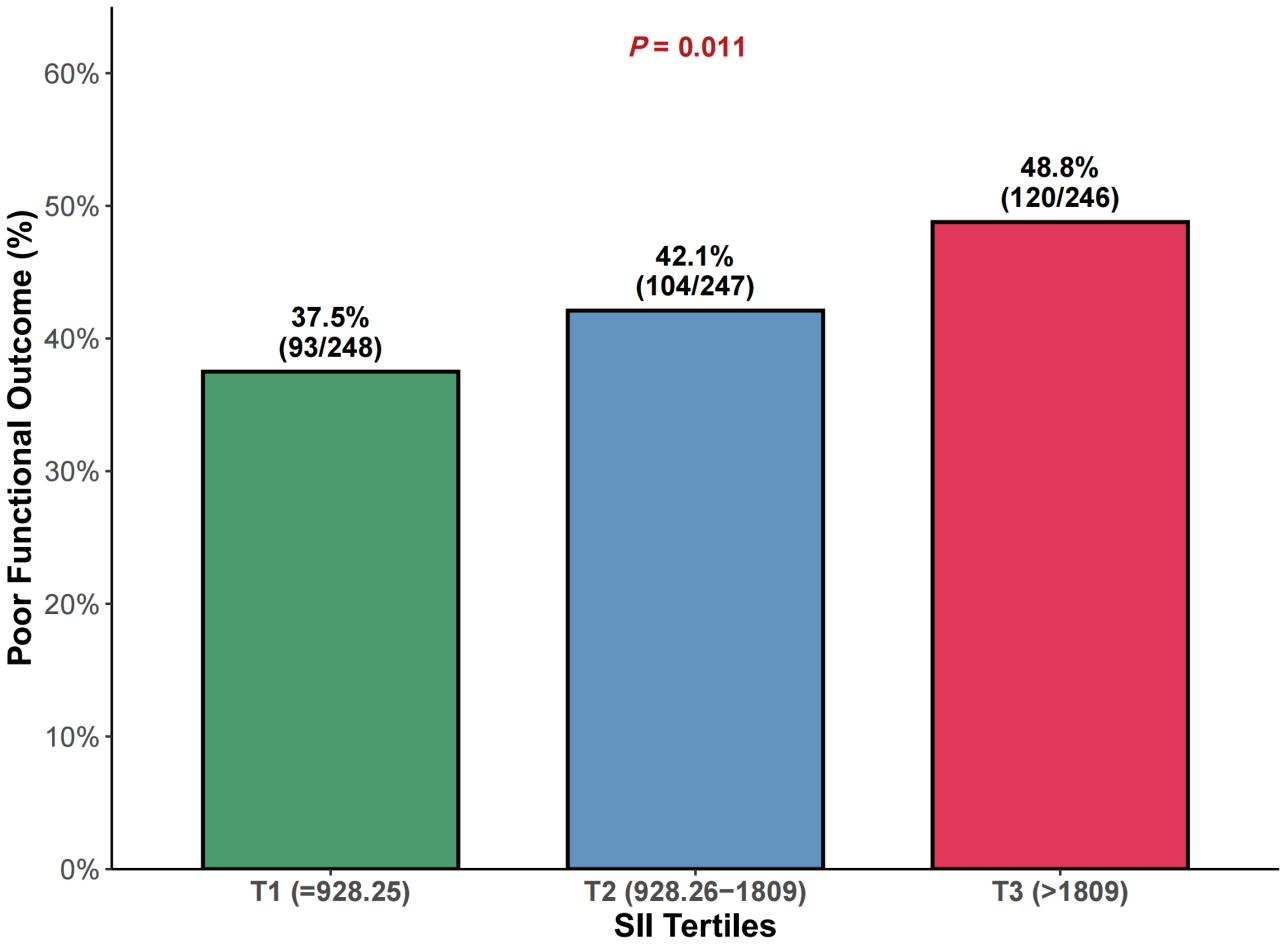
Comparison of poor functional outcome (mRS 3–6) rates across SII tertiles at 3 months. Proportion of patients with poor functional outcome (mRS 3–6) at 3 months across systemic immune-inflammation index (SII) tertiles. *P* value represents the chi-square test for trend. SII tertile ranges: T1 ≤ 928.25 (*n* = 247), T2 928.26–1,809 (*n* = 247), T3 >1,809 (*n* = 247). mRS, modified Rankin Scale; SII, systemic immune-inflammation index.

Poor functional outcome (mRS 3–6) at 3 months occurred in 317 patients (42.8%) overall, with rates increasing across SII tertiles: 37.2% (92/247) in T1, 42.5% (105/247) in T2, and 48.6% (120/247) in T3 (*P* = 0.011; Figure 2).

In multivariable analysis (Table 2), log-transformed SII as a continuous variable was significantly associated with poor functional outcome. The association remained significant across all models: unadjusted (OR 1.38, 95% CI 1.13–1.68, *P* = 0.002), age and sex-adjusted (OR 1.45, 95% CI 1.18–1.78, *P* < 0.001), and fully adjusted (OR 1.428, 95% CI 1.159–1.759, *P* < 0.001).

**Table 2:** Association between SII and poor functional outcome (mRS 3–6) at 3 months in patients with anterior circulation acute ischemic stroke undergoing endovascular thrombectomy.

**Categories**	**Model 1**		**Model 2**		**Model 3**	
	**OR (95%CI)**	***P*** **- value**	**OR (95%CI)**	***P*** **- value**	**OR (95%CI)**	***P*** **- value**
Log (SII) continuous	1.38 (1.13–1.68)	0.002^**^	1.45 (1.18–1.78)	< 0.001^***^	1.428 (1.159–1.759)	< 0.001^***^
Tertiles						
T1	Ref		Ref		Ref	
T2	1.25 (0.87–1.79)	0.233	1.29 (0.89–1.87)	0.172	1.25 (0.86–1.84)	0.243
T3	1.59 (1.11–2.28)	0.011^*^	1.75 (1.21–2.53)	0.003^**^	1.73 (1.18–2.52)	0.005^**^
Pfortrend		0.011^*^		0.003^**^		0.005^**^

^*^P < 0.05, ^**^P < 0.01, ^***^P < 0.001. Model 1: Unadjusted model. Model 2: Adjusted for age and gender. Model 3: Fully adjusted model - adjusted for age, gender, smoking, hypertension, diabetes, atrial fibrillation, thrombolysis, ASPECT score, and puncture to reperfusion time. **SII tertile ranges:** T1: ≤ 928.25 (*n* = 247), T2: 928.26–1,809 (*n* = 247), T3: >1,809 (*n* = 247). Log (SII) represents natural logarithm-transformed SII values analyzed as a continuous variable due to the right-skewed distribution of SII. SII, systemic immune-inflammation index; OR, odds ratio; CI, confidence interval; mRS, modified Rankin Scale; ASPECT, Alberta Stroke Program Early CT Score.

When analyzed by tertiles, compared to the lowest tertile (T1), patients in T3 had significantly higher odds of poor functional outcome in all models. In the fully adjusted model, the OR for T3 was 1.73 (95% CI 1.18–2.52, *P* = 0.005), while T2 showed no significant difference (OR 1.25, 95% CI 0.86–1.84, *P* = 0.243). The P for trend across tertiles was significant (*P* = 0.005), confirming a dose-response relationship.

Sensitivity analyses including baseline NIHSS (Model 4) demonstrated directionally consistent associations with attenuated effect estimates, as theoretically predicted when adjusting for a mediator (Supplementary Table S1). For continuous log-transformed SII, the adjusted OR was 1.29 (95% CI 1.04–1.60, *P* = 0.023). When analyzed by tertiles, compared to T1, the adjusted OR for T3 was 1.39 (95% CI 0.93–2.06, *P* = 0.107), though the trend test across tertiles was no longer statistically significant (*P* for trend = 0.107). This pattern of effect attenuation while maintaining directional consistency supports our theoretical framework and confirms that our primary analysis (Model 3) better estimates the total effect of SII on functional outcomes.

### Subgroup and interaction analyses

**Figure 3:**
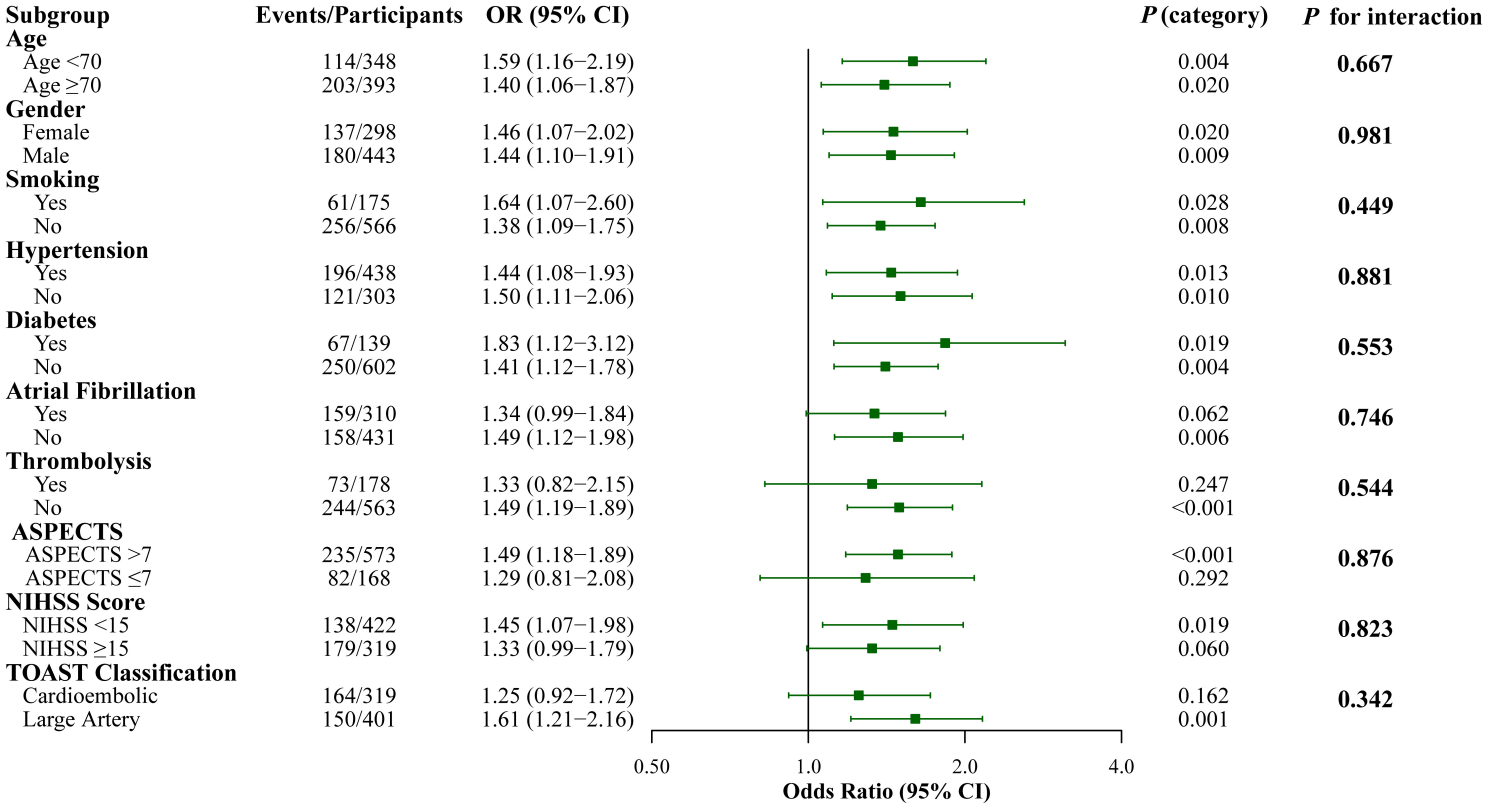
Subgroup Analysis of the Association Between SII and Poor Functional Outcome (mRS 3–6) at 3 months. Forest plot showing the association between log-transformed SII (continuous variable) and poor functional outcome (mRS 3–6) across different subgroups. Each row displays the odds ratio (95% CI) and individual *P*-value for that specific subgroup, along with the *P* for interaction testing whether the SII-outcome association differs significantly between subgroup categories. All analyses were adjusted for age, gender, smoking, hypertension, diabetes, atrial fibrillation, thrombolysis, ASPECTS, and puncture to reperfusion time.

Subgroup analyses demonstrated generally consistent associations across patient characteristics (Figure 3). Individual subgroup analyses showed varying levels of statistical significance, with some subgroups not reaching significance due to reduced sample sizes after stratification. However, no significant interactions were detected (all *P* for interaction > 0.05), indicating that the prognostic value of SII was consistent across different patient populations regardless of age, gender, comorbidities, stroke severity, or treatment modalities.

### Restricted cubic spline analysis

**Figure 4:**
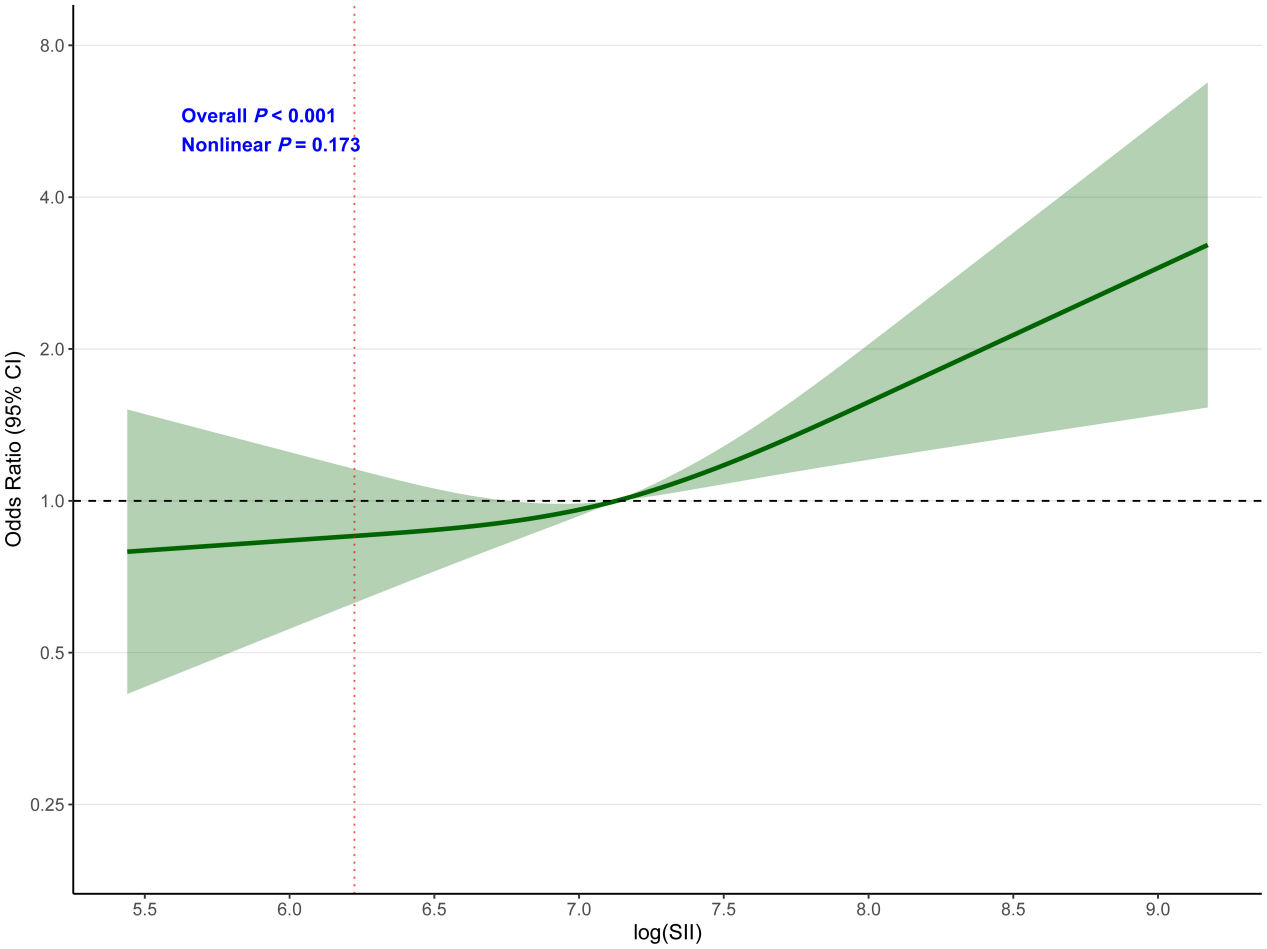
Dose-response relationship between SII and poor functional outcome (mRS 3–6) at 3 months. Restricted cubic spline analysis showing the association between log(SII) and poor functional outcome (mRS 3–6) at 3 months. The model was adjusted for age, gender, smoking, hypertension, diabetes, atrial fibrillation, thrombolysis, ASPECTS, and puncture to reperfusion time. The solid line represents the odds ratio, and the shaded area represents the 95% confidence interval. Reference value: 10th percentile (log_SII = 6.224). Overall *P* < 0.001; *P* for non-linearity = 0.173.

Restricted cubic spline analysis (Figure 4) demonstrated a significant overall association between log (SII) and poor functional outcome (overall *P* < 0.001), with no evidence of non-linearity (*P* for non-linearity = 0.173). This indicates a linear relationship between increasing log (SII) values and the odds of poor functional outcome throughout the observed range.

### Safety outcome: symptomatic intracranial hemorrhage

Symptomatic intracranial hemorrhage (sICH) occurred in 39 patients (5.3%) overall, with rates of 5.3% (13/247) in T1, 3.2% (8/247) in T2, and 7.3% (18/247) in T3 (*P* = 0.148; Table 3).

**Table 3:** Association between SII and symptomatic intracranial hemorrhage in patients with anterior circulation acute ischemic stroke undergoing endovascular thrombectomy.

**Categories**	**Model 1**		**Model 2**		**Model 3**	
	**OR (95%CI)**	***P*** **-value**	**OR (95%CI)**	***P*** **-value**	**OR (95%CI)**	***P*** **-value**
Log (SII) continuous	1.24 (0.81–1.89)	0.329	1.27 (0.83–1.94)	0.275	1.32 (0.84–2.07)	0.235
Tertiles						
T1	Ref		Ref		Ref	
T2	0.60 (0.25–1.48)	0.269	0.61 (0.25–1.51)	0.286	0.56 (0.22–1.42)	0.222
T3	1.42 (0.68–2.95)	0.356	1.47 (0.70–3.07)	0.311	1.50 (0.70–3.21)	0.295
*P* for trend		0.315		0.278		0.260

Model 1: Unadjusted model. Model 2: Adjusted for age and gender. Model 3: Fully adjusted model - adjusted for age, gender, smoking, hypertension, diabetes, atrial fibrillation, thrombolysis, ASPECT score, and puncture to reperfusion time. **SII tertile ranges:** T1: ≤ 928.25 (*n* = 247), T2: 928.26–1,809 (*n* = 247), T3: >1,809 (*n* = 247). SII, systemic immune-inflammation index; OR, odds ratio; CI, confidence interval; sICH, symptomatic intracranial hemorrhage; ASPECT, Alberta Stroke Program Early CT Score.

Neither log (SII) as a continuous variable nor SII tertiles showed significant associations with sICH in any of the regression models. The fully adjusted OR for log (SII) was 1.32 (95% CI 0.84–2.07, *P* = 0.235). No significant trends were observed across tertiles (*P* for trend = 0.260).

## Discussion

This study comprehensively evaluated the association between admission SII levels and clinical outcomes in 741 patients with anterior circulation acute ischemic stroke undergoing endovascular thrombectomy. To ensure homogeneity and enhance the internal validity of our findings, we specifically focused on anterior circulation strokes, which have the most robust evidence base for EVT efficacy (2, 3, 9). Our main findings demonstrate that: (1) elevated SII was independently associated with poor functional outcome at 3 months (adjusted OR 1.428, 95% CI 1.159–1.759), with a clear dose-response relationship; (2) SII showed no association with hemorrhagic complications in our anterior circulation cohort; (3) the prognostic value of SII remained consistent across all examined subgroups without significant interactions; and (4) the relationship between log(SII) and poor functional outcome was linear throughout the observed range.

The median SII value in our cohort (1,247.9 [IQR: 804.9–2,127.1]) was comparable to previous studies in EVT-treated patients (19–23). The SII integrates three key components of the systemic inflammatory response: neutrophils (innate immunity and tissue damage), lymphocytes (adaptive immunity and neuroprotection), and platelets (thrombosis and inflammation) (13). This composite index may better capture the complex interplay between inflammation and thrombosis than single-cell ratios (14).

The mechanisms underlying the association between elevated SII and poor functional outcomes likely involve multiple interconnected pathways. Neutrophilia reflects an enhanced inflammatory response that can exacerbate reperfusion injury through the release of reactive oxygen species, proteolytic enzymes, and neutrophil extracellular traps (NETs) (32). These NETs not only contribute to microvascular thrombosis but also directly damage the blood-brain barrier (32). Lymphopenia, conversely, indicates immunosuppression and reduced neuroprotective capacity, increasing susceptibility to infections and impairing tissue repair mechanisms (33). Elevated platelet counts may contribute to the “no-reflow” phenomenon despite successful macrovascular recanalization, as activated platelets promote microthrombosis and amplify inflammatory cascades through platelet-leukocyte interactions (34). This multifaceted inflammatory response ultimately leads to secondary brain injury beyond the initial ischemic insult (35).

Our finding of a linear dose-response relationship, confirmed by restricted cubic spline analysis (*P* for non-linearity = 0.173), supports a direct biological gradient between inflammatory burden and clinical outcomes. This linear relationship suggests that SII could be used as a continuous biomarker rather than requiring categorical cutoffs, enhancing its clinical utility and allowing for more nuanced risk stratification.

A notable finding was the lack of association between SII and symptomatic intracranial hemorrhage, the most clinically relevant safety endpoint. This finding contrasts with some previous studies that reported associations between inflammatory markers and hemorrhagic transformation (18, 21). Several factors may explain this discrepancy. First, our exclusive focus on anterior circulation strokes may represent a more homogeneous population with different hemorrhagic risk profiles compared to mixed cohorts. Second, our post-procedural SII measurement captures the inflammatory response after intervention rather than baseline inflammation (16), and sICH is primarily influenced by mechanical factors, reperfusion dynamics, and anticoagulation management rather than systemic inflammation alone. Third, the differential inflammatory pathways may explain why SII is associated with functional outcomes but not bleeding risk. Inflammation contributes to ischemic injury progression, whereas hemorrhagic complications depend more on endothelial integrity and reperfusion dynamics than systemic inflammatory burden (36).

The consistency of SII's prognostic value across all subgroups strengthens its potential clinical applicability. We found no significant interactions with age, sex, comorbidities, stroke severity, or treatment modalities, suggesting that SII could be incorporated into risk stratification models without requiring subgroup-specific adjustments. The absence of interaction with thrombolysis indicates that SII maintains its prognostic value regardless of bridging therapy, supporting its use in both direct and bridging thrombectomy protocols. Similarly, the consistent effect across TOAST classifications supports SII as a general prognostic marker rather than an etiology-specific indicator in anterior circulation strokes. This uniform effect across diverse patient characteristics within our homogeneous anterior circulation cohort suggests that post-procedural inflammatory response may be more related to infarct size and reperfusion injury than to specific patient characteristics or stroke etiology.

Our findings have several potential clinical implications. First, SII represents a readily available biomarker calculated from routine laboratory tests. The observed association between higher SII tertiles and poor outcomes suggests that SII may contribute to risk stratification, though further validation is needed. Second, the absence of association with hemorrhagic complications in our cohort suggests that elevated SII alone may not necessitate modification of antithrombotic therapy, though clinical decisions should be individualized. Third, the association between elevated SII and poor functional outcomes through inflammatory pathways warrants further investigation into whether anti-inflammatory interventions might benefit high-SII patients.

Our variable selection strategy, guided by DAG principles, prioritized estimation of the total effect of SII by avoiding adjustment for potential mediators such as baseline NIHSS. Sensitivity analyses including NIHSS demonstrated the expected pattern of effect attenuation while maintaining directional consistency, supporting our causal framework and ensuring transparency of results. This approach allows our findings to capture all pathways through which inflammatory response influences functional outcomes, providing the most clinically relevant prognostic information.

This study has several strengths, including a relatively large sample size from a single center (ensuring treatment consistency), comprehensive outcome assessment including both efficacy and safety endpoints, robust statistical methodology with multiple sensitivity analyses, and a homogeneous cohort focused on anterior circulation strokes. However, several limitations merit consideration. First, the retrospective single-center design may limit generalizability. Second, single time-point SII measurement fails to capture the dynamic inflammatory response. Third, our focus on anterior circulation strokes, while enhancing internal validity, may limit generalizability to posterior circulation strokes. Fourth, excluding unsuccessful recanalization cases may have introduced selection bias. Additionally, our focus on hemorrhagic complications rather than infection-related outcomes such as stroke-associated pneumonia represents a limitation, as the latter may be more mechanistically related to systemic inflammation. Stroke-associated pneumonia information was not systematically collected in our retrospective database with sufficient standardization for reliable analysis. Future prospective studies should examine SII associations with both hemorrhagic and infectious complications, particularly stroke-associated pneumonia, to provide a more comprehensive safety profile and better understand the relationship between SII and various post-stroke complications including functional outcomes.

Future research should address these limitations through prospective multicenter studies with serial SII measurements, longer follow-up periods, and inclusion of both anterior and posterior circulation strokes to assess whether our findings are territory-specific. Correlation with specific inflammatory biomarkers and neuroimaging markers of inflammation could further elucidate underlying mechanisms. Randomized trials testing anti-inflammatory interventions in high-SII patients could establish whether this association is modifiable.

## Conclusions

In patients with anterior circulation acute ischemic stroke undergoing endovascular thrombectomy, elevated SII is independently associated with poor functional outcome but not symptomatic intracranial hemorrhage. The consistent prognostic value across diverse patient subgroups and linear dose-response relationship support SII as a robust, clinically applicable biomarker for this specific population. These findings suggest that SII assessment may provide additional prognostic information in anterior circulation EVT-treated patients, though prospective validation is warranted.

## Data Availability

The raw data supporting the conclusions of this article will be made available by the authors, without undue reservation.
