# Supplementary material for: Systemic immune-inflammation index associated with functional outcomes after endovascular thrombectomy in anterior circulation acute ischemic stroke
Source: Front Neurol. 2025 Oct 22;16:1663299. doi: 10.3389/fneur.2025.1663299 (PMC12587767; doi:10.3389/fneur.2025.1663299)
Supplement: Supplementary file 1 [file Table_1.docx]

|  | **Categories** | **Model 4** |  |
| --- | --- | --- | --- |
|  |  | **OR (95%CI)** | ***P* - value** |
|  | **Log (SII) continous** | 1.29 (1.04-1.60) | 0.023* |
|  | **Tertiles** |  |  |
|  | **T1** | Ref |  |
|  | **T2** | 1.16 (0.78-1.73) | 0.460 |
|  | **T3** | 1.39 (0.93-2.06) | 0.107 |
|  | ***P* for trend** |  | 0.107 |

**Table S1. Association Between SII and Poor Functional Outcome (mRS 3-6) at 3 Months in Patients with Anterior Circulation Acute Ischemic Stroke Undergoing Endovascular Thrombectomy**

**P* < 0.05

Model 4: Adjusted for age, gender, smoking, hypertension, diabetes, atrial fibrillation, thrombolysis, ASPECT score, puncture to reperfusion time, and admission NIHSS score.

**SII tertile ranges:** T1: ≤928.25 (n=247), T2: 928.26-1809 (n=247), T3: >1809 (n=247).

Log (SII) represents natural logarithm-transformed SII values analyzed as a continuous variable due to the right-skewed distribution of SII.

**Abbreviations:** SII, systemic immune-inflammation index; OR, odds ratio; CI, confidence interval; mRS, modified Rankin Scale; ASPECT, Alberta Stroke Program Early CT Score.
